# Preliminary experience with continuous right ventricular pressure and transesophageal echocardiography monitoring in orthotopic liver transplantation

**DOI:** 10.1371/journal.pone.0263386

**Published:** 2022-02-04

**Authors:** Lachlan F. Miles, Etienne J. Couture, Cristhian Potes, Timothy Makar, Malindra C. Fernando, Akshay Hungenahally, Matthew D. Mathieson, Hannah Perlman, Marcos V. Perini, Dilraj Thind, Laurence Weinberg, André Y. Denault

**Affiliations:** 1 Department of Critical Care, The University of Melbourne, Melbourne, Australia; 2 Department of Anaesthesia, Austin Health, Melbourne, Australia; 3 Division of Intensive Care Medicine, Department of Anesthesiology and Department of Medicine, Quebec Heart and Lung Institute, Laval University, Quebec City, Canada; 4 Edwards LifeSciences Pty. Ltd., Irvine, California, United States of America; 5 Department of Surgery, The University of Melbourne, Melbourne, Australia; 6 Victorian Liver and Intestinal Transplant Unit, Austin Health, Melbourne, Australia; 7 Department of Anesthesiology and Critical Care Division, Montreal Heart Institute, Université de Montréal, Montréal, Canada; Scuola Superiore Sant’Anna, ITALY

## Abstract

**Background:**

Despite increasing attention in the cardiac anesthesiology literature, continuous measurement of right ventricular pressure using a pulmonary artery catheter has not been described in orthotopic liver transplantation, despite similarities in the anesthetic approach to the two populations. We describe our preliminary experience with this technique in orthotopic liver transplantation, and by combining various derived measures with trans-esophageal echocardiography, make some early observations regarding the response of these measures of right ventricular function during the procedure.

**Methods:**

In this case series, ten patients (five men and five women) undergoing orthotopic liver transplantation in our institution had their surgeries performed while monitored with a pulmonary artery catheter with continuous right ventricular port transduction and trans-esophageal echocardiography. We recorded various right ventricular waveform (early-to-end diastolic pressure difference, right ventricular outflow tract gradient, right ventricular dP/dT and right ventricular end-diastolic pressure) and echocardiographic (right ventricular fractional area change, tricuspid annular plane systolic excursion, right ventricular lateral wall strain) and described their change relative to baseline at timepoints five minutes before and after portal vein reperfusion, immediately after hepatic artery reperfusion and on abdominal closure.

**Results:**

Except for tricuspid annular plane systolic excursion at five minutes prior to reperfusion (mean −0.8 cm; 95% CI−1.4, –0.3; p = 0.007), no echocardiographic metric was statistically significantly different at any timepoint relative to baseline. In contrast, changes in right ventricular outflow tract gradient and right ventricular dP/dt were highly significant at multiple timepoints, generally peaking immediately before or after reperfusion before reducing, but not returning to baseline in the neohepatic phase. Nine of 10 participants in this series demonstrated a degree of dynamic right ventricular outflow tract obstruction, which met criteria for hemodynamic significance (> 25 mmHg) in two participants. These changes were not materially affected by cardiac index.

**Conclusions:**

Dynamic right ventricular outflow tract obstruction of varying severity appears common in patients undergoing orthotopic liver transplantation. These results are hypothesis generating and will form the basis of future prospective research.

## Introduction

The surgical insult of orthotopic liver transplantation (OLT)–coupled with the consequences of end-stage liver disease–increase the potential for major blood loss and fluid shifts, making it a high-risk surgical procedure. Hemodynamic perturbation during OLT is common, particularly around the time of reperfusion. Such instability is associated with operative morbidity and mortality, and has an incidence of 12–77% depending on the case series reviewed [1, 2].

The pathophysiology of hemodynamic instability during OLT (separate to that which is due to major hemorrhage) is mutifactorial, with severity of the pre-operative liver disease, electrolyte derangement, ischemia-reperfusion injury of the donor allograft, cirrhotic cardiomyopathy, and hyperdynamic circulation all implicated. Additionally, acute decompensation of right ventricular (RV) function or ventriculo-arterial decoupling has been postulated to contribute to hemodynamic instability [3, 4]. Studies examining transesophageal echocardiographic metrics have shown that the RV is vulnerable during OLT, with 22% of cases demonstrating isolated RV dysfunction immediately after reperfusion [5].

Recently, continuous RV pressure (RVP) monitoring via an appropriately equipped pulmonary artery catheter (PAC) has gained a new appreciation in the cardiac anesthesiology community. This technique can provide: (1) a direct visualization of RV systolic and diastolic function, (2) diagnose dynamic RV outflow tract obstruction, and (3) inform the requirement for and the effect of inhaled pulmonary vasodilator therapy [6–8]. However, this monitoring technique has not been studied previously in OLT, despite similarities in the anesthetic approach to these two populations. We aim to initially address this evidentiary gap by reporting our preliminary experience with continuous RVP monitoring in a series of 10 patients undergoing OLT in our institution, combining the results with continuous TEE metrics. Our objective was to record our observations and identify possible end points for future prospective work. This case series describes for the first time in the literature (insofar as we are aware) the changes in these measures over the course of the procedure.

## Methods

This study was approved by the human research ethics committee of our institution, which included a waiver of informed consent (Audit/19/Austin/69). The use of a PAC and TEE during OLT are considered the standard of care in our institution. Patients were excluded from the study if they had: (1) an absolute contraindication to intra-operative TEE (i.e., grade III esophageal varices, severe portal hypertensive gastropathy, gastrointestinal hemorrhage within the preceding 30 days, or previous major esophageal surgery), (2) had a starting heart rhythm other than sinus rhythm (as this would interfere with the capture and offline analysis of TEE loops), or (3) were less than 165 cm tall (as there was a perceived risk that the balloon of the PAC would wedge in the pulmonary artery (PA) prior to the RV port being correctly positioned). Participants were enrolled between 19 November 2019 and 30 August 2020.

### Intra-operative monitoring

**Fig 1:**
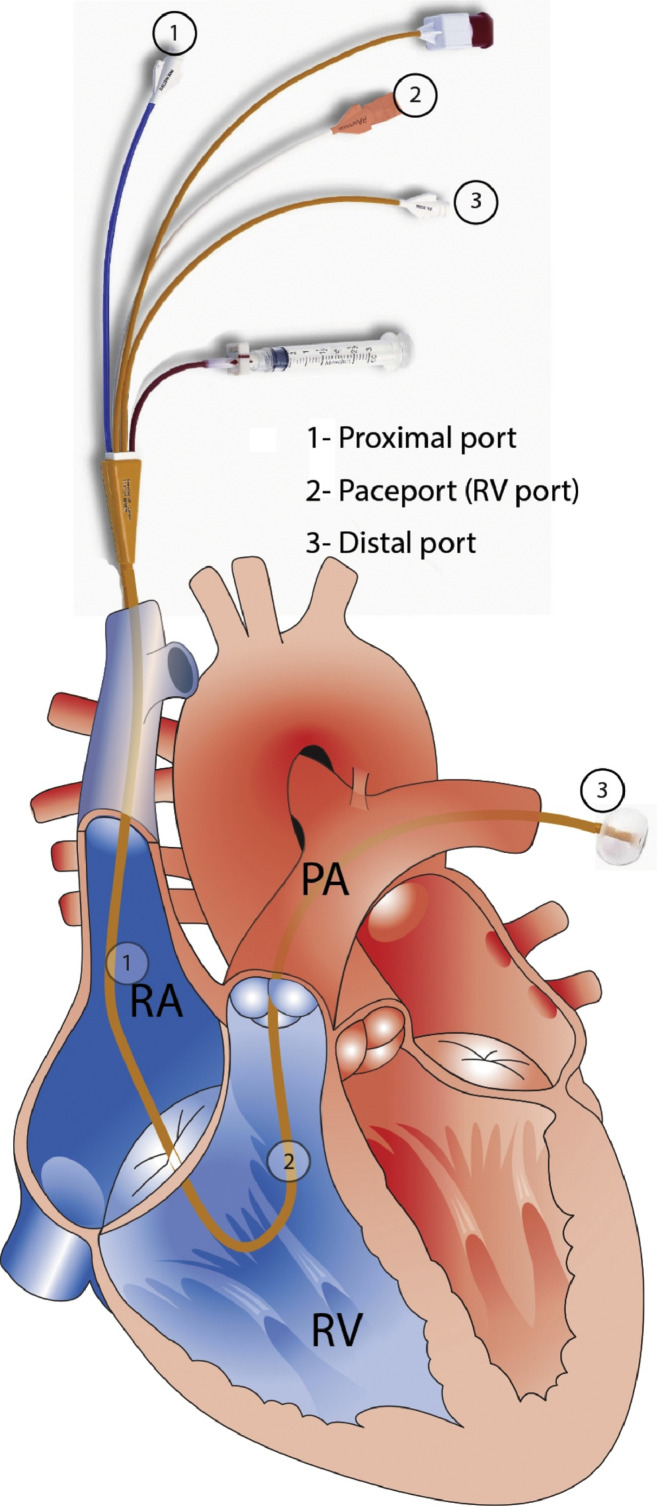
Schematic representation of the position of a pulmonary artery catheter capable of right ventricular pressure monitoring inside the right heart (Thermodilution Paceport Pulmonary Artery Catheter, product reference 931F75, Edwards Lifesciences, Irvine, CA). PA, pulmonary artery; RA, right atrium; RV, right ventricle. From Raymond M, Grønlykke L, Couture EJ, et al. Perioperative Right Ventricular Monitoring in Cardiac Surgery. J Cardiothorac Vasc Anes 2019;33:1090–104. Figure used with permission.

Following the induction of anesthesia, a PAC with inbuilt central venous pressure (CVP), RVP and pulmonary artery pressure ports were inserted into the right internal jugular vein (VIP+ PAC, Edwards Lifesciences Pty. Ltd., Irvine, CA) with the tip positioned in the PA and the RV port (which was set 19 cm back from the tip of the PAC) positioned in the RV (Fig 1). The technique for the correct positioning of this catheter has been previously described (8). A radial and femoral arterial line were also inserted. A TEE probe was placed (x7-2t or x8-2t probe with an iE33 or EPIQ CVx ultrasound system, Philips, Eindhoven, Netherlands) prior to commencement of the surgery. All patients received underbody convection warming and an indwelling urinary catheter. Continuous cardiac output was either measured using model-simulated cardiac output (FloTrac, Edwards LifeSciences Pty. Ltd., Irvine, CA) during the case, or calculated using the same technology using the arterial waveform and offline processing. This technique has been shown to provide a reliable measure of cardiac output during liver transplantation relative to bolus thermodilution [9], provides a continuous measurement and thus more accurate trend data, and does not require the anesthesiologist to manually perform the measurement.

### Anesthesia and surgical management

The same induction technique (using propofol [1.5 – 2 mg/kg], fentanyl [3 – 5 μg/kg] and suxamethonium [1 mg/kg]) was used in all the patients in this study. Anesthesia was maintained with sevoflurane or isoflurane in an air/oxygen mixture, with additional infusions of fentanyl and cisatracurium. Metaraminol, norepinephrine, and vasopressin were administered according to the anesthesiologist’s preference. All patients received an end-to-side caval anastomosis during their transplant with partial inferior vena cava clamping. This technique is preferred in our institution as it has been shown to better preserve portocaval flow (and thus venous return and cardiac output) relative to veno-venous bypass or total caval cross-clamping [10]. Reperfusion was supported in all patients with boluses of calcium chloride and metaraminol.

### Data collection and offline analysis

A variety of recipient characteristics were collected, including age, sex, the pre-operative model for end-stage liver disease (MELD) score, height, weight, body surface area, body mass index, and indication for transplant. Conventional symbols were used to denote different phases of the liver transplant: ‘I’ is the prehepatic or dissection phase; ‘II’ is the anhepatic phase, which is defined by the clamping of the portal vein, removal of the native liver, implantation of the donor allograft, and unclamping of the portal vein; and ‘III’ is the neohepatic or post-reperfusion phase of the liver transplant.

A series of six-beat TEE loops were captured at predefined time points (as described by Fukazawa et al. [11]). Specifically, these were:

- At commencement of the operation (i.e., the baseline)
- Five minutes prior to portal vein reperfusion (i.e., III – 5)
- Five minutes after portal vein reperfusion (i.e., III + 5)
- On hepatic artery (HA) reperfusion
- 240 minutes after portal vein reperfusion or at wound closure, whichever time point was reached first (i.e., III + 240).

Mid-esophageal four-chamber TEE images were acquired intra-operatively and stored for subsequent offline analysis of RV function. Speckle tracking echocardiography was used to obtain the tricuspid annular plane systolic excursion (TAPSE), RV fractional area change (RV FAC), and RV longitudinal strain. Peak and end-systolic strain was obtained for basal, mid, and apical segments of the RV lateral and septal walls. Lateral, septal, and global strain was obtained using the mean longitudinal strain value of the individual segments forming the wall of interest. End-systolic and end-diastolic RV dimensions were also obtained. The post-processing analysis was completed using EchoInsight software (Epsilon Imaging, Ann Arbor, MI).

**Fig 2:**
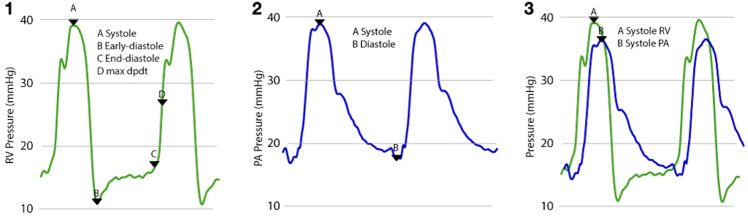
Schematic representation of right ventricular pressure (⎼⎼⎼) and pulmonary artery pressure (⎼⎼⎼) waveforms showing (1) the derivation of the right ventricular metrics of RV systolic pressure, RV early diastolic pressure, RV end diastolic pressure and the point of maximum dP/dt; (2) the derivation of the pulmonary artery metrics of PA systolic and PA end diastolic pressures; and (3) the overlapped waveforms showing peak RV and PA systolic pressures. The difference between these two pressures forms the RVOT gradient. Acronyms: max dP/dt, maximum rate of change in pressure over change in time; PA, pulmonary artery RV, right ventricular; RVOT, right ventricular outflow tract.

Waveforms and pressures were displayed continuously during the procedure (Intellivue MX800 monitor, Philips, Eindhoven, Netherlands) and captured for further offline analysis using open-access software [12]. While these waveform data were captured for the entire procedure, only the 60 seconds on either side of the predefined time points were analyzed. Specific RV waveform metrics analyzed at each time point were the RV change in pressure over time (dP/dt), peak gradient across the RV outflow tract (RVOT) gradient derived by subtracting the PA systolic pressure from the RV systolic pressure, the RV early-to-end-diastolic pressure difference, and RV end diastolic pressure (Fig 2).

### Statistical analysis

Given the exploratory nature of this analysis, we did not undertake a formal sample size calculation. Instead, our arbitrary series size of 10 participants was determined *a priori*. Data are presented as mean (and its corresponding standard deviation [SD]) or median (the corresponding interquartile range [IQR]) depending on normality. Statistical comparisons were performed comparing the mean (at the 95% confidence interval [95% CI]) change in echocardiographic or waveform parameters at each time point (Baseline, III – 5, III + 5, HA, and III + 240) relative to the baseline. A linear effects model was chosen because our data featured repeated sampling and some missing data [13]. The Kenward-Roger method was selected to minimize type I errors, given the small sample size [14]. Parameters of RV function and cardiac index were individually modelled on a fixed effect of time points and a random intercept of patient identification. These changes were represented using the standard units of measurement for each variable except for the RV early-to-end-diastolic pressure difference and RV diastolic pressure. The models for these latter two variables suffered from heteroskedasticity of variables and were therefore reparametrized with a logarithmic transformation to correct a moderate right skew. Heteroskedasticity was no longer present on subsequent re-analysis. Given this requirement for logarithmic transformation, the results are presented as percentage change from the baseline. Finally, to remove the effects of confounding factors from the interpretation of RV dP/dt and gain an impression of the effects of the procedure on RV contractility in isolation, the RV systolic pressure (i.e., as a surrogate for afterload), RV end-diastolic pressure (i.e., as a surrogate for preload), and heart rate were added to the linear effects model for this metric. Statistical comparisons were considered significant when p < 0.05.

## Results

Ten patients, comprising five men and five women, were monitored using the described technique. The median age of the study sample was 56.5 years (IQR 46.8–62.7 years). The median duration of the operation was 497 minutes (IQR 461–519 minutes), and the mean MELD score was 19 points (SD 11). Specific participant characteristics are shown in Table 1. Of the 50 possible time points examined, eight (16%) needed to be excluded because of RV catheter displacement, which was usually caused by the RV port straddling the tricuspid valve and thereby delivering an unreliable waveform. Satisfactory catheter positioning in Patient 1 could not be achieved at any time point, leading to the exclusion of waveform-derived data for this participant.

**Table 1:** Participant characteristics.

Patient	Sex	Age (years)	Height (cm)	Weight (kg)	MELD	Indication	Duration of surgery (minutes)
1	Female	68	168	101	12	NASH	522
2	Male	41	196	124	7	HCC	548
3	Male	60	172	75	8	HCC	495
4	Female	44	165	58	20	PSC	508
5	Female	55	172	115	25	NASH	690
6	Male	67	176	86	24	PSC	500
7	Male	55	168	58	7	HCC	480
8	Female	57	165	62	42	AIH	395
9	Female	31	174	63	28	Alcohol	350
10	Male	63	178	80	20	Alcohol	454

Acronyms: Autoimmune hepatitis (AIH); hepatocellular carcinoma (HCC); model for end-stage liver disease score (MELD); non-alcoholic steatohepatitis (NASH); primary sclerosing cholangitis (PSC).

### Echocardiographic variables

The results of the analysis for changes in echocardiographic parameters are shown in Table 2. No metric reached statistical significance except for a significant reduction in TAPSE at the III – 5 time point (−0.8 cm [95% CI −1.4, −0.3]; p = 0.007).

**Table 2:** Differences in echocardiographic metrics at each study time point.

**RV FAC (%)**	**Mean (SD)**	**Mean (95% CI) difference**	***p* value**
Baseline	40 (11)		
III – 5	35 (12)	−5.2 (−13.3, 2.9)	0.20
III + 5	37 (14)	−3.3 (−11.4, 4.8)	0.41
HA	38 (10)	−2.3 (−10.4, 5.8)	0.57
III + 240	37 (14)	−3.2 (−11.3, 4.9)	0.43
**RV lateral wall strain (%)**	**Median (IQR)**	**Mean (95% CI) difference**	***p* value**
Baseline	–25 [–28, –22]		
III – 5	–21 [–26, –15]	3.6 (−1.8, 9.0)	0.19
III + 5	–21 [–32, –18]	−0.1 (−5.5, 5.3)	0.97
HA	–26 [–31, –20]	−0.5 (−5.7, 4.9)	0.85
III + 240	–25 [–33, –15]	0.7 (−4.7, 6.1)	0.80
**TAPSE (cm)**	**Median (IQR)**	**Mean (95% CI) difference**	***p* value**
Baseline	2.8 [2.0, 3.5]		
III – 5	1.4 [1.1, 2.5]	−0.8 (−1.4, –0.3)	0.007
III + 5	2.2 [1.6, 3.2]	−0.3 (−0.9, 0.3)	0.25
HA	2.5 [2.1, 3.1]	−0.2 (−0.7, 0.4)	0.62
III + 240	2.8 [1.6, 3.8]	0.1 (−0.5, 0.7)	0.78

Acronyms: Hepatic artery (HA); right ventricular fractional area change (RV FAC); tricuspid annular plane systolic excursion (TAPSE).

### Waveform variables

The analysis results for changes in waveform parameters are shown in Table 3. Cardiac index was elevated significantly relative to baseline at the III + 5 timepoint (0.8 [0.1–1.5]; p = 0.025) and at III + 240 (1.3 [0.6–2.0]; p < 0.001). While there was a general trend towards progressive increases in cardiac index over the course of the procedure, observed increases were not significant at the other timepoints. Meaningful differences in RV dP/dt were observed across multiple time points, with increases compared to baseline (mean 309 mmHg/s, SD 79) observed following reperfusion at the III + 5 (169 mmHg/s increase [95% CI 114, 225]; p < 0.001) at HA (96 mmHg/s increase [95% CI 41, 152]; p = 0.001), and at III + 240 (142 mmHg/s increase [95% CI 86, 197]; p < 0.001) time points. Note that the normal RV dP/dt is > 400 mmHg/s [15]. Similarly, the gradient across the RVOT was increased at all time points relative to the baseline (median 6.6 mmHg, IQR 4.8–12.3), with an initial increase at III – 5 (5.2 mmHg increase [95% CI 2.0, 8.3]; p = 0.002) and further sustained increases following reperfusion at III + 5 (10.5 mmHg increase [95% CI 7.6, 13.4]; p < 0.001), HA (6.8 mmHg increase [95% CI 3.9, 9.7]; p < 0.001), and III + 240 (7.9 mmHg increase [95% CI 4.9, 10.9]; p < 0.001). The elevation in RVOT gradient was largely unchanged even after statistical correction for cardiac index and norepinephrine at all timepoints (Table 4). The RV end-diastolic pressure was also lower relative to the baseline (median 9.5 mmHg, IQR 8.5–16.7) at III – 5 (–27% [95% CI −45, −4]; p = 0.026), but statistical significance was not reached at any other time point. In contrast, the early-to-end-diastolic pressure difference was not significantly altered statistically relative to the baseline (4.3 mmHg, IQR 3.2–7.0) at any time point other than at III – 5 (−30% reduction [−46, −9]; p = 0.01).

**Table 3:** Differences in waveform metrics at each study time point.

**CI (L/min/m** ^**2**^ **)**	**Mean (SD)**	**Mean (95% CI) difference**	**p-value**
Baseline	3.3 (1.0)		
III– 5	3.3 (1.0)	0.04 (−0.6, 0.7)	0.9
III + 5	4.0 (1.3)	0.8 (0.1, 1.5)	0.025
HA	3.8 (1.2)	0.6 (−0.1, 1.2)	0.11
III + 240	4.6 (1.4)	1.3 (0.6, 2.0)	< 0.001
**RV dP/dt (mmHg/s)**	**Mean (SD)**	**Mean (95% CI) difference**	**p-value**
Baseline	309 (79)		
III – 5	330 (81)	35 (30, 100)	0.28
III + 5	477 (137)	168 (106, 230)	< 0.001
HA	406 (99)	97 (35, 159)	0.003
III + 240	458 (122)	151 (84, 219)	< 0.001
**RVOT gradient (mmHg)**	**Mean (SD)**	**Mean (95% CI) difference**	**p-value**
Baseline	7.1 (3.5)		
III – 5	12.9 (5.9)	5.2 (2, 8.3)	0.002
III + 5	17.6 (6.6)	10.5 (7.6, 13.4)	< 0.001
HA	14 (6.5)	6.8 (3.9, 9.7)	< 0.001
III + 240	15.6 (4.7)	7.9 (4.9, 10.9)	< 0.001
**RVEDP (mmHg)**	**Median (IQR)**	**Mean (95% CI) % change**	**p-value**
Baseline	9.5 [8.5, 16.7]		
III – 5	8.5 [4.8, 9.2]	−27 [−45, −4]	0.026
III + 5	10.4 [6.2, 15.1]	−11 [−33, 18]	0.41
HA	8.2 [7.5, 11.6]	−15 [−36, 13]	0.25
III + 240	11.0 [8.2, 15.6]	−8 [−31, 22]	0.55
**Early-to-end-diastolic pressure difference (mmHg)**	**Median (IQR)**	**Mean (95% CI) % change**	**p-value**
Baseline	4.2 [3.2, 7.0]		
III – 5	3.7 [2.7, 3.8]	–30 (–46, –9)	0.01
III + 5	5.0 [4.0, 6.1]	10 (–16, 43)	0.48
HA	4.2 [3.3, 5.1]	–4 (–26, 25)	0.79
III + 240	5.8 [4.5, 6.4]	24 (–5, 61)	0.11

Acronyms: Cardiac index (CI); change in pressure over time (dP/dt); hepatic artery (HA); right ventricular (RV); right ventricular end-diastolic pressure (RVEDP); right ventricular outflow tract (RVOT).

**Table 4:** Effects of cardiac index and norepinephrine dose on the measurement of RVOT gradient.

RVOT gradient (mmHg)	Mean (95% CI) change compared to baseline uncorrected	p-value	Mean (95% CI) change compared to baseline corrected for cardiac index	p-value	Mean (95% CI) change compared to baseline corrected for norepinephrine dose	p-value
III– 5	5.2 (2.0, 8.3)	0.002	5.3 (2.1, 8.5)	0.002	5.6 (1.7, 9.5)	0.007
III + 5	10.5 (7.6, 13.4)	< 0.001	10.2 (7.0, 13.3)	< 0.001	11.2 (6.4, 16.0)	< 0.001
HA	6.8 (3.9, 9.7)	< 0.001	6.6 (3.5, 9.6)	< 0.001	7.4 (2.9, 12.0)	0.002
III + 240	7.9 (4.9, 10.9)	< 0.001	7.2 (3.5, 10.9)	< 0.001	8.4 (4.4, 12.3)	< 0.001

Acronyms: RVOT, right ventricular outflow tract.

The correction of dP/dt for other hemodynamic metrics–the RV systolic pressure (i.e., the afterload), RV diastolic pressure (i.e., the preload) and heart rate—is shown in Table 5. For every 1 mmHg increase in RV systolic pressure, dP/dt was observed to increase by 8 mmHg/s (95% CI 5, 11; p < 0.001). However, following correction for this surrogate measure, dP/dt was still shown to independently increase (together with RVOT gradient) at all time points following reperfusion (III + 5, +101 mmHg/s [95% CI 51, 152], p < 0.001; HA, +61 mmHg/s [95% CI 15, 106], p = 0.01; and III + 240, +76 mmHg/s [95% CI 26, 126], p = 0.04). Neither RV end-diastolic pressure (p = 0.45) nor heart rate (p = 0.16) were observed to significantly improve the fit of the model.

**Table 5:** Effect of confounding hemodynamic factors on the measurement of RV dP/dt.

Measurement	Mean effect (95% CI) on dP/dt per single unit increase	p value
RV peak systolic pressure (mmHg)	8 (5, 11)	< 0.001
RV end-diastolic pressure (mmHg)	2 (−4, 8)	0.45
Heart rate (beats/minute)	1.6 (−0.7, 3.9)	0.16

Acronyms: Right ventricular (RV); change in pressure over change in time (dP/dt).

No patient in this study sustained any major complications, defined as PA rupture from PAC insertion or an esophageal (i.e., variceal) injury from TEE.

Data outlining norepinephrine dose for each patient at each time point and total fluid volume and type administered over the course of each procedure are contained in S1 Appendix. The minimal data set to replicate all study findings is contained in S2 Appendix.

## Discussion

This is the first prospective exploratory observational study investigating the effects of OLT on echocardiographic and pressure waveform metrics of RV function. These were assessed using predefined time points relevant to hemodynamic instability [11]. While statistical significance was not achieved across most metrics, certain trends suggest some measures may be valid endpoints in confirmatory studies, particularly the metrics derived from continuous RVP waveform analysis. Additionally, the procedure appears to result in a statistically significant increase in the gradient across the RVOT (independent of cardiac index) as well as an increased RV dP/dt, possibly reflecting a decrease in pulmonary vascular elastance and dynamic RVOT obstruction. Dynamic RVOT obstruction has been reported previously in lung transplantation following implantation of an allograft with normal pulmonary vasculature—in a native RV that had previously been exposed to chronically elevated pulmonary vascular resistance [16–19]. However, given the small sample size of this study and the exploratory nature of these analyses, these findings can be considered hypothesis generating at best. Additionally, this hypothesis cannot be proven definitively without the ability to continuously measure PA elastance (3). Nevertheless, the improvement in the dP/dt model fit after correcting for RV systolic pressure as a surrogate for afterload, combined with a worsening gradient across the RVOT makes this theory biologically plausible, and the observed increase in dP/dt could be a normal biological response (that is, an Anrep effect) [20]. This theory is supported by the observed relationship between dP/dt and RV peak systolic pressure. Alternatively, the described phenomenon could be reflective of a primary increase in the inotropic state. While this might explain an increase in gradient at the III + 5 time point (i.e., immediately after the routine administration of calcium chloride), it does not explain the persistent elevation of this metric during the remainder of the neohepatic period, as use of inotropes beyond reperfusion was limited in this sample. Furthermore, the observed increase in RVOT gradient was statistically independent of the similar observed increase in cardiac index.

Hemodynamic instability is common during OLT (particularly around the time of reperfusion), and is multifactorial [1]. The existing series in the literature suggest a possible contribution of RV dysfunction to this phenomenon. In 1993, De Wolf et al. examined various metrics in a series of 20 patients using a PAC equipped with a fast response thermistor that derived RV ejection fraction, RV end-systolic volume index, and RV end-diastolic volume index [21]. The authors reported RV function to be generally “well preserved” throughout the procedure, as we too observed, with the only abnormality detected with this device being a “small and probably clinically unimportant decrease in RV ejection fraction during the anhepatic stage.” The surgery was performed using veno-venous bypass (as opposed to the partial caval clamp technique that is routinely employed in our institution), which may have reduced hemodynamic perturbation. In another study, Xu et al. (using a similarly modified PAC) examined the RV ejection fraction in 30 patients undergoing OLT using complete caval cross-clamp and reported a progressive deterioration in this metric between the baseline and III + 5, with the RV ejection fraction recovering to the baseline by the end of the procedure [4]. A similar phenomenon may have been reflected in the statistically significant reduction in TAPSE that we observed at the III – 5 time point. However, the accuracy of measures of cardiac output derived through continuous and intermittent thermodilution has been called into question, especially around reperfusion, possibly due to increased thermal noise, a phenomenon highlighted by Böttiger et al. [22]. A subsequent study by the same group comparing three-dimensional echocardiographic reconstruction of the RV showed that this technique was similarly inaccurate (i.e., underestimating the stroke volume), although, in contrast to their previous work, this series suggested that comparator thermodilution cardiac output measures were acceptable [23]. Consequently, these authors were unable to determine which monitoring technique would be better to monitor the RV during OLT (i.e., three-dimensional TEE or thermodilution cardiac output).

The techniques employed by the aforementioned studies derived metrics of RV performance and right sided cardiac output indirectly, as opposed to the technique (particularly RVP waveform analysis) employed in this study, which has the benefit of measuring RV hemodynamic parameters directly. While direct transduction of the RV waveform using a PAC with an appropriately positioned RV lumen is increasingly employed in cardiac anesthesiology [8, 24], it has not been described during OLT. Additionally, the effects of the various phases of the procedure on the RVOT gradient, dP/dt, or early-to-end-diastolic pressure difference have similarly not been described. Of particular interest is the detection of varying degrees of dynamic RVOT obstruction (defined as a RVOT gradient > 6 mmHg) in 9 out of 10 participants in our study at various time points. In two participants, the RVOT gradient at III + 5 exceeded the threshold for hemodynamic significance (> 25 mmHg) [25]. However, the frequency with which this phenomenon occurred in our sample suggests it might be a previously unrecognized finding in this population. However, more significant gradients in cardiac surgery and lung transplantation have been associated with major hemodynamic compromise, termed (rather evocatively) ‘suicide RV’ [17].

We acknowledge some limitations. Firstly, despite the prospective nature of this investigation, the small sample size means that the findings cannot be considered robust. However, it must be emphasized that the purpose of this study was not to definitively describe the functional response of the RV during OLT but to determine whether a confirmatory study was warranted and to inform the design of any larger confirmatory trial. Hence, irrespective of the high levels of statistical significance demonstrated in some metrics, all the results of this study are considered hypothesis generating rather than definitive. Secondly, maintenance of a stable RV pressure waveform could not be maintained in all patients for the duration of the OLT (as evidenced by the exclusion of data from Patient 1). It is hypothesized that the large fluid shifts that accompany this procedure can result in dynamic changes in RV size at various times during the procedure, and that despite the catheter position remaining constant during the procedure the position of the tricuspid annulus may not be as stable. Accordingly, if the RV lumen is located only a short distance beyond the valve (as may occur in shorter patients since the RV port is some 19 cm proximal to the end of the catheter), it may move between the right atrium and the RV, depending on the volume state of the chamber. This occurred despite a deliberate attempt to exclude shorter patients (i.e., those < 165 cm tall) from the study. Thirdly, the attending anesthesiologist was unblinded; that is, the anesthesiologist had access to the RV waveform. While the RV pressure waveform analysis is not routinely used in our center, there was no guarantee that a treating clinician has not acted on any abnormality observed (particularly in the two patients who developed hemodynamically significant RVOT obstruction), thereby altering some of the metrics recorded. Finally, the intravenous volume administered to the patients relative to each time point was not recorded, mainly because of the difficulty in determining the preload from this metric alone. Additionally, we did not obtain metrics that would have allowed the derivation of pulmonary vascular resistance (i.e., pulmonary artery occlusion pressure). Routine wedging of the PAC balloon is not part of our routine practice due to the perceived risk of pulmonary artery rupture. However, such information would be of use to further delineate changes in the pulmonary vasculature over the course of OLT, and other authors may wish to consider including this measurement in future studies if (1) it is part of their routine practice; or (2) if participants are consented to this specific risk.

This study records our preliminary experience using direct RV pressure monitoring and TEE to examine various measures of RV performance during OLT. Our exploratory statistical analysis suggests that certain waveform-derived metrics studied would be of use in further confirmatory work. Additionally, and unexpectedly, the results indicate that varying degrees of dynamic RVOT obstruction may be common in patients undergoing OLT, that this may worsen over the course of the procedure without a return to the baseline, and that is affected minimally by any change in cardiac index. It is hypothesized that decreased pulmonary vascular elastance due to end-stage liver disease and donor allograft reperfusion are potential drivers of this phenomenon, although confirmatory studies are required. Despite obtaining a high degree of statistical significance for this metric, the small sample size of this study prevents any definitive conclusions from being drawn. Larger prospective studies using these monitoring modalities are necessary to investigate these findings further to quantify the effects of OLT on RV function and potentially examine the contribution of RV dysfunction and dynamic RVOT obstruction to hemodynamic disturbance during OLT in more detail.

## Supporting information

S1 Appendix Intraoperative intravenous volume and norepinephrine requirements for study participants. (DOCX)

S2 Appendix Deidentified echocardiographic and waveform metrics at each timepoint for study participants. (XLSX)
