# Supplementary material for: Preliminary experience with continuous right ventricular pressure and transesophageal echocardiography monitoring in orthotopic liver transplantation
Source: PLoS One. 2022 Feb 4;17(2):e0263386. doi: 10.1371/journal.pone.0263386 (PMC8815904; doi:10.1371/journal.pone.0263386)
Supplement: S1 Appendix — (DOCX) [file pone.0263386.s001.docx]

**S1 Appendix**

*Table 1.* Intravenous fluid totals administered over the course of the primary operation to participants.

|  | **Allogeneic RBC (mL)** | **Autologous RBC (mL)** | **Donor whole blood (mL)** | **Platelets (mL)** | **FFP (mL)** | **Cryoprecipitate (mL)** | **Other colloid* (mL)** | **Total (mL)** |
| --- | --- | --- | --- | --- | --- | --- | --- | --- |
| Case 1 | 3064 | 7650 | 1125 | 1468 | 550 | 1800 | 22900 | 38557 |
| Case 2 | 521 | 2400 | 800 | 0 | 0 | 0 | 7504 | 11225 |
| Case 3 | 0 | 900 | 0 | 367 | 0 | 0 | 6100 | 7900 |
| Case 4 | 1459 | 1125 | 0 | 0 | 0 | 0 | 5346 | 7930 |
| Case 5 | 2430 | 3150 | 0 | 734 | 825 | 660 | 5670 | 13469 |
| Case 6 | 0 | 0 | 0 | 0 | 825 | 600 | 2400 | 3825 |
| Case 7 | 2060 | 3060 | 0 | 367 | 1100 | 660 | 11720 | 18967 |
| Case 8 | 1522 | 1195 | 0 | 734 | 550 | 300 | 3000 | 7301 |
| Case 9 | 1070 | 2624 | 0 | 367 | 550 | 0 | 7200 | 11811 |
| Case 10 | 3126 | 4216 | 0 | 1101 | 2750 | 900 | 14500 | 26593 |

Acronyms: RBC, red blood cells; FFP, fresh frozen plasma. * 4% albumin solution.

*Table 2.* Norepinephine doses (μg/kg/minute) at each timepoint for each participant involved in the study.

| **Timepoint** | **Case 1** | **Case 2** | **Case 3** | **Case 4** | **Case 4** | **Case 6** | **Case 7** | **Case 8** | **Case 9** | **Case 10** |
| --- | --- | --- | --- | --- | --- | --- | --- | --- | --- | --- |
| Baseline | 0.01 | 0 | 0 | 0.03 | 0.01 | 0 | 0 | 0.08 | 0.03 | 0.01 |
| III – 5 | 0.1 | 0.03 | 0.08 | 0.24 | 0.13 | 0.12 | 0.05 | 0.19 | 0.16 | 0.18 |
| III + 5 | 0.2 | 0.08 | 0.13 | 0.34 | 0.23 | 0.23 | 0.14 | 0.10 | 0.24 | 0.23 |
| HA | 0.15 | 0.06 | 0.12 | 0.34 | 0.17 | 0.17 | 0.09 | 0.13 | 0.32 | 0.23 |
| III + 240 | 0.15 | 0.06 | 0.11 | 0.38 | 0.17 | 0.02 | 0.09 | 0.13 | 0.08 | 0.19 |
